# Intercalation of Dyes in Graphene Oxide Thin Films and Membranes

**DOI:** 10.1021/acs.jpcc.1c00327

**Published:** 2021-03-23

**Authors:** Andreas Nordenström, Nicolas Boulanger, Artem Iakunkov, Igor Baburin, Alexey Klechikov, Alexei Vorobiev, Alexandr V. Talyzin

**Affiliations:** † Department of Physics, Umeå University, S-90187 Umeå, Sweden; ‡ Theoretische Chemie, Technische Universitat Dresden, Bergstraße 66b, 01062 Dresden, Germany; § Department of Physics and Astronomy, Uppsala University, Uppsala 751 20, Sweden

## Abstract

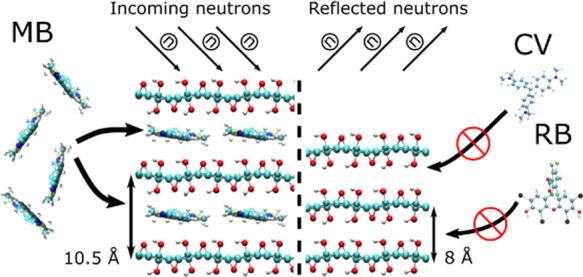

Intercalation of dyes into thin multilayered graphene oxide (GO) films was studied by neutron reflectivity and X-ray diffraction. Methylene blue (MB) penetrates the interlayer space of GO in ethanol solution and remains intercalated after the solvent evaporation, as revealed by the expansion of the interlayer lattice and change in chemical composition. The sorption of MB by thin GO films is found to be significantly stronger compared to the sorption of Crystal violet (CV) and Rose bengal (RB). This effect is attributed to the difference in the geometrical shape of planar MB and essentially nonflat CV and RB molecules. Graphite oxides and restacked GO films are found to exhibit different methylene blue (MB) sorptions. MB sorption by precursor graphite oxide and thin spin-coated films of GO is significantly stronger compared to freestanding micrometer-thick membranes prepared by vacuum filtration. Nevertheless, the sorption capacity of GO membranes is sufficient to remove a significant part of the MB from diluted solutions tested for permeation in several earlier studies. High sorption capacity results in strong modification of the GO structure, which is likely to affect permeation properties of GO membranes. Therefore, MB is not suitable for testing size exclusion effects in the permeation of GO membranes. It is not only hydration or solvation diameter but also the exact geometrical shape of molecules that needs to be taken into account considering size effects for penetration of molecules between GO layers in membrane applications.

## 1 Introduction

Graphene oxide (GO) is a two-dimensional (2D) material prepared by delamination of graphite oxides in polar solvents. The dispersions can be used for deposition of multilayered materials composed of randomly restacked GO sheets. Drop-casting^1^ and spin-coating^2^ are the methods most commonly used for preparation of GO thin films, while vacuum filtration is used to prepare freestanding membranes.^3,4^ Multilayered graphene oxide laminates in the form of papers,^5^ thin films,^6^ or membranes^1,7,8^ have attracted much attention over the past 8 years, with first reports dating back to the 1960s.^9^ Membranes have been proposed for various applications, e.g., gas separation,^10^ nanofiltration,^9,11,12^ separation of solvent mixtures,^13,14^ as a toxicant barrier,^15^ and in water desalination.^16^ In the following, films and membranes formed by restacking of many GO layers will be named GO films and GO membranes, respectively.

Most of the applications cited above are enabled by the ability of GO multilayers to swell in polar solvents, similar to their precursor graphite oxides.^17−19^ Swelling of precursor graphite oxides in polar solvents was studied in detail over the past 150 years,^20^ most typically in water^21,22^ and alcohols.^23−26^ The swelling of graphite oxides depends on the synthesis method, most notably for the materials prepared by Brodie’s and Hummers’s oxidation routes.^26−28^ Recent studies revealed also that the swelling of GO membranes can be significantly different compared to precursor graphite oxides.^3,4^ The extensive overlap of single-layered GO flakes in restacked multilayered structures results in a zigzag-like pathway for diffusion of solvent molecules and for ions or molecules dissolved in a solvent.^9^ The thicker the membrane, the longer the diffusion pathway for ions and molecules. Therefore, slower kinetics of sorption and change in the overall sorption can be expected for molecules and ions capable of penetrating between GO flakes.^29^ However, most of the sorption studies to date have been performed using bulk graphite oxides.

Swelling of GO membranes is directly related to the size of “permeation channels” provided by the interlayer space, which enable diffusion of solvents and solutions. The interlayer distance of the GO structure is typically studied using X-ray diffraction (XRD),^22,30,31^ direct measurements of film/membrane thickness,^32^ or quantitative evaluation of solvent vapor sorption.^29,33,34^ Simultaneous evaluation of GO film thickness and the amount of adsorbed solvent can be performed using neutron reflectivity (NR) methods.^2,35^

Swelling of the GO structure provides the possibility for dissolved ions and molecules to enter the interlayer space and to be adsorbed in significant amounts. Therefore, sorption of ions and molecules can be used as a test instrument to verify the GO interlayer distance in solutions.^36^ It is particularly interesting to check if molecules of common dyes can penetrate into the GO interlayer space. For example, the size of hydrated/solvated methylene blue (MB) ions has been considered in several studies to be larger than the size of permeation channels, thus preventing diffusion across the membrane.^37−39^ However, the high sorption capacity of graphite oxides toward dyes was not taken into account in these studies.

This is surprising since it is well-known that graphite oxides demonstrate a high sorption capacity for MB.^40^ Moreover, sorption of MB was used in several studies for the evaluation of the accessible surface area of GO in aqueous solutions.^41−43^ The different sorption capacities of pristine and defected GO are likely to be a reason for the rather large discrepancy in reported gravimetric values for MB sorption by GO in the literature, typically 250–450 mg/g,^40,41^ but with some reports up to ≈700 mg/g^44^ with a maximal value of 870 mg/g for extremely defected GO.^45^

Most commonly, the dye sorption tests of GO are performed in water-dispersed single- or few-layered states.^44,46−48^ The GO flakes dispersed in water will adsorb MB using the whole surface without hindrance provided by the diffusion in the interlayer space. The tests performed using bulk graphite oxide powders are also often limited to the quantitative estimation of sorption with little information about the intercalation and change of structure.^47^ The results obtained for bulk graphite oxides cannot be directly related to GO membranes and thin films. Several examples demonstrate a strong difference between sorption properties of restacked GO multilayers and bulk graphite oxide powders.^3,4^ However, a direct quantitative estimation of sorption in thin films is difficult.

Recently, we demonstrated that neutron reflectivity (NR) can be used as a powerful method to study sorption of polar solvents reversibly intercalated into GO thin films from vapors.^2,49,35^ Here, we study the sorption and intercalation of dyes by thin GO films immersed in liquid solutions using NR and XRD. The dye sorption was compared for three types of solid GO materials: submicrometer-thick thin films, freestanding few-micrometer-thick membranes, and bulk graphite powders. Smaller sorption of MB is found for freestanding micrometer-thick membranes as compared to thin films and bulk powders. XRD and NR experiments demonstrate that MB ions penetrate into the interlayer space in ethanol solution and intercalate the GO structure with significant lattice expansion. Therefore, the absence of MB and some other dyes’ diffusion across the GO membranes is not related to the size exclusion effect but connected to the direct chemical modification of the membrane structure.

## 2 Materials and Methods

Graphite oxide was synthesized following the slightly modified Hummers procedure as described in details elsewhere.^28^ Precursor graphite oxide with C/O = 2.5 and sulfur content 0.34 at % (as found by XPS; see Figure S5) was used for preparation of GO dispersions. The graphite oxide powder was sonicated for 12 h in an ethanol/water mixture (90% ethanol by volume) and centrifuged at 4400 rpm for 60 min, yielding a dispersion with a concentration of approximately 1 mg/mL. The GO solution was deposited onto clean 5 × 5 cm^2^ Si substrates using spin-coating followed by drying at ambient conditions for several days. Spin-coating was performed using 1000 rpm for 1 min followed by 1900 rpm for 10 s. The acceleration rate of 500 rpm/s was used for the first step and 2000 rpm/s for the second step. The procedure was repeated 4–7 times using ≈0.5 mL of solution manually dropped over the surface of the substrate. The resulting GO films are almost transparent with a slight brownish color. The typical thickness in the ambient condition ranges from 300 to 600 Å depending on the number of dropping cycles. The GO films can be considered as dense layered materials with layers oriented parallel to the substrate. The hydrophilic nature of GO along with its flexibility prevents the formation of voids as a significant fraction of the total film volume.^50^ GO films prepared using a very similar procedure have been extensively characterized in our previous studies for the sorption of solvents and solvent mixtures from the vapor phase.^2,35^ The films selected for NR characterization exhibited a uniform thickness (except for corners of the rectangular substrate) and a smooth surface.^35^

Freestanding GO membranes (5–6 μm) were prepared by vacuum filtration of water dispersions with typical GO concentration 2 mg/mL. The precursor Hummers GO by Abalonyx, Oslo, Norway, was used for the membrane preparation. C/O for the precursor GO was 2.2 (excluding oxygen from sulfate impurities) as found by XPS. Note that the precursor GO for the membranes was slightly different from that of the thin films.

Neutron reflectivity experiments were performed at the reflectometer SuperADAM at the Institute Laue–Langevin (ILL), Grenoble, France, using a monochromatic neutron beam with wavelength 5.19 Å. The fitting of neutron data was made using BoToFit software. X-ray diffraction (XRD) patterns were recorded using a Panalytical X’pert X-ray diffractometer with Cu Kα radiation (λ = 1.5418 Å). The XRD data were recorded at ambient air humidity conditions, which were observed to stay within 22–51% from day to day.

## 3 Results and Discussion

### 3.1 Sorption of Dyes by GO Thin Films from Ethanol Solution

**Figure 1:**
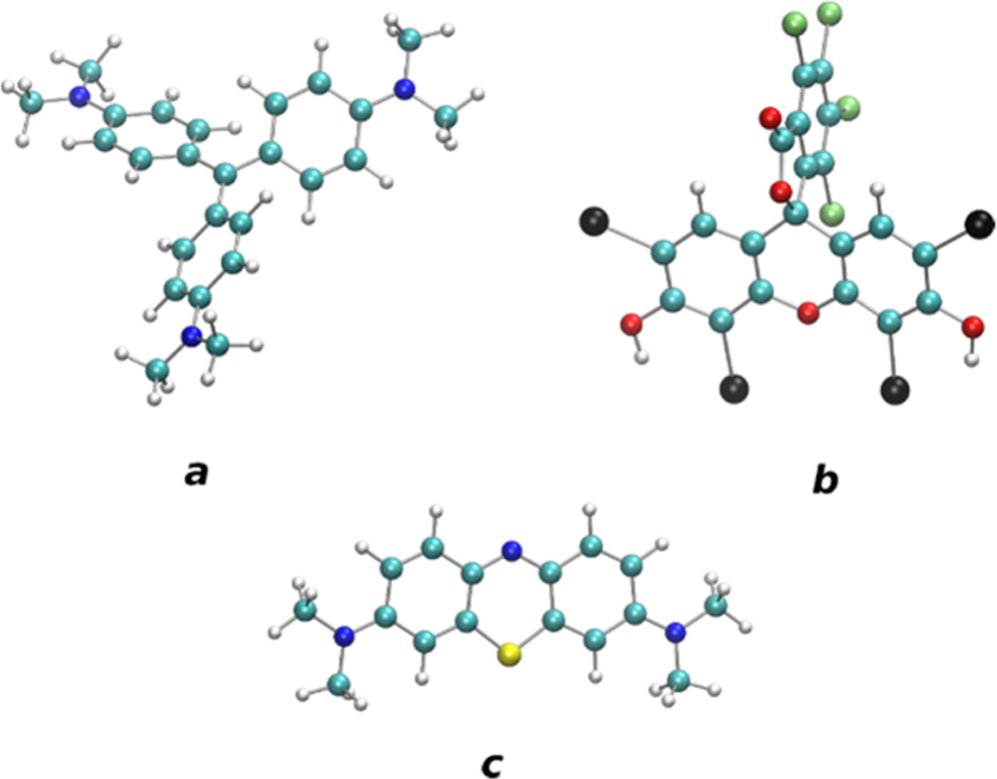
Molecular structure of dyes tested for sorption by GO in this study: (a) crystal violet (C_25_N_3_H_30_Cl), (b) Rose bengal (C_20_H_2_C_l4_I_4_Na_2_O_5_), and (c) methylene blue (C_16_H_18_C_l_N_3_S). Note that only methylene blue has a flat geometry.

Thin GO films were deposited on the Si substrate using spin-coating, air-dried for several days, and studied by the NR method prior to and after exposure to an ethanol solution consisting of three dyes: methylene blue (MB), Rose bengal (RB), and crystal violet (CV) (Figure 1).

**Figure 2:**
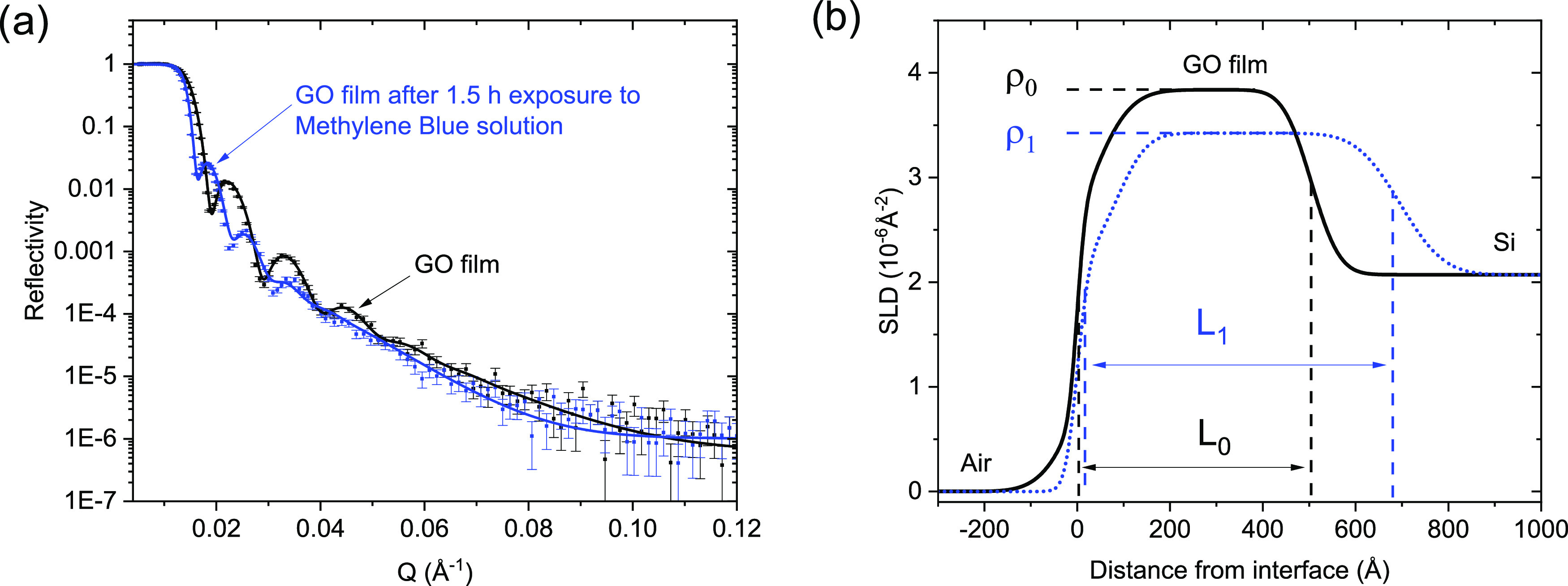
(a) Neutron reflectivity data (including error bars) obtained from the GO film in ambient conditions before and after MB sorption. (b) Scattering length density profile of the GO film before and after MB sorption obtained as a result of the modeling (fitting) of experimental data.

The GO films were immersed into an ethanol solution of dyes for 1.5 h, and the change in film thickness and chemical composition due to sorption/intercalation of dyes was evaluated using NR at ambient air conditions. The NR curves recorded from the precursor GO film before and after MB sorption are shown in Figure 2. The reflectometry curve shows well-defined oscillations typical for high-quality GO films with homogeneous thickness (Figure 2a). The NR curve recorded from the GO film after exposure to the MB solution is clearly different from the pristine GO film, reflecting an increase in the film thickness and change in the chemical composition. According to our previously published studies, the GO film swells in ethanol, which rapidly and reversibly evaporates from the film exposed to air conditions.^2,3,35^

Therefore, the change in the film thickness and composition after removal of excess solution and drying can be assigned to MB sorption. Analysis of NR data allows one to evaluate the change in film thickness and to calculate the sorption of MB by the GO film. Assuming that the film is composed of GO flakes parallel to the substrate, it is also possible to estimate the change in the interlayer distance (*d*) using the change in film thickness. Fitting of the NR data for the pristine GO film allows one to calculate the scattering length density (SLD) parameter, which is related to the chemical composition of the film (3.84 × 10^–6^ Å^–2^), and the SLD profile providing film thickness (501 Å); see Figure 2b.

Using the interlayer distance value of 7.9 Å determined using XRD, the film was estimated to consist of 63.4 layers. Assuming that water-free GO has the composition C_2_O_0.8_H_0.24_, the experimentally observed SLD value also allows one to calculate the amount of water absorbed by the film at ambient conditions (≈40% humidity) to be 0.67 water molecules per formula unit of GO, resulting in the overall formula C_2_O_0.8_H_0.24_ + (H_2_O)_0.67_.

The reference experiment was performed to verify the stability of the GO film in liquid ethanol. The GO film was characterized by NR in the pristine state and after immersion in pure ethanol for 1.5 and 24 h. Analysis of data showed that the ethanol is reversibly removed from the GO film by air-drying (see details in the SI file). Thickness change <18 Å was detected in both reference tests, which provides error ±0.3 Å in the evaluation of *d*-spacing. The error is likely related to small day-to-day variations of humidity (within 30–40%) in the experimental hall. In the following calculations, we assume that the number of GO layers does not change after immersion in the ethanol solution of dyes.

**Figure 3:**
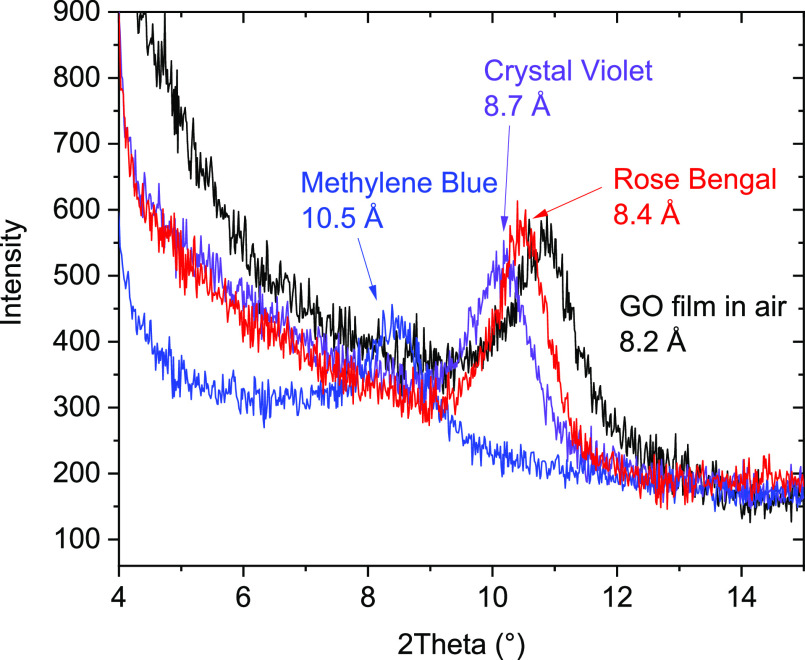
X-ray diffraction patterns recorded at ambient air conditions from the pristine GO film and GO films after sorption of methylene blue, Rose bengal, and crystal violet.

Fitting the NR data recorded from the GO film exposed to concentrated MB solution and air-drying allows one to evaluate the increase in the interlayer distance and to evaluate the amount of adsorbed MB. Assuming that the number of GO layers is unchanged after exposure of the sample to ethanol solution of dyes and after air-drying, the increase in the film thickness can be assigned to the sorption of dye molecules. The increase of the GO film thickness from 501 to 683 Å corresponds to an increase of the *d*-value from 7.9 to 10.8 Å, and the change in SLD (from 3.84 × 10^–6^ to 3.42 × 10^–6^ Å^–2^) corresponds to the sorption of 0.043 molecules of MB per formula unit (see the SI file for details of calculations). Calculated in gravimetric units, this corresponds to a sorption of 279 mg of MB per gram of GO. The interlayer distance *d* = 10.8 Å value found for the GO/MB film is in reasonable agreement with the experimental *d*(001) = 10.5 Å value recorded using XRD (Figure 3) considering that ambient humidity was not precisely controlled in our experiments and changed within 10–15%.

It should be noted that the value obtained using XRD takes into account only the change in the interlayer distance of crystalline domains, whereas the thickness of the film can be affected also by packing of layers and other imperfections. For example, the thickness of the film will be higher if the GO layers are not exactly parallel to the substrate or there are voids between lamellas. As a result of the disorder, the thickness of the GO film could be, in principle, somewhat larger compared to the thickness calculated using the interlayer distance provided by XRD. Therefore, the good agreement between the values of the interlayer distance determined using XRD and NR demonstrates the high quality of the film and the nearly perfect parallel orientation of GO flakes.

Similar sorption experiments were performed with GO films exposed to ethanol solutions of Rose bengal (RB) and crystal violet (CV). The results are summarized in Table 1; see also the SI file for complete NR data. The changes in the GO film thickness and composition due to the sorption of RB appeared to be smaller compared to the sorption of MB. The film thickness increased from 530 to 564 Å, while the change in the SLD value corresponds to the sorption of 0.004 molecules per GO formula unit (80 mg RB/gGO). Relatively small sorption was observed also for CV. The thickness of the GO film increased from 380 to 436 Å, which corresponds to an increase in the interlayer distance from 7.9 to 9.1 Å. The change in SLD corresponds to a sorption of 0.018 mol/f.u or 151 mg CV/gGO. A rather small increase in *d*(001) was also observed in XRD patterns recorded from samples of GO films after RB and CV sorption.

**Table 1:** Summary of Most Important Parameters Found by NR Experiments^a^

dye name	*L*_0_ (Å)	*L*_1_ (Å)	molecules per f.u.	sorption (mg/g)
methylene blue	501	683	0.043	279
crystal violet	380	436	0.018	151
Rose bengal	530	564	0.004	80

a L0 and L1 refer to thicknesses of the GO film before and after sorption of the dye, respectively.

**Figure 4:**
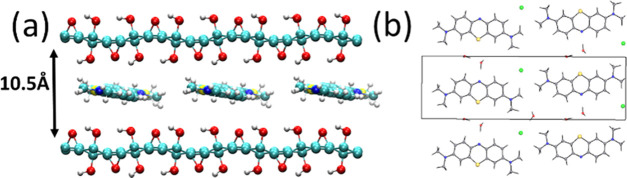
Schematic view of the GO structure intercalated by methylene blue: (a) structure of the interlayer intercalated by the MB molecules; (b) top view of the close-packed MB layer (as in the crystal structure of the solid MB).

It can be concluded that NR results provide firm evidence for penetration of MB into the GO interlayer space in ethanol solution causing an expansion of the GO lattice. The increase in the interlayer distance correlates well with the size of the MB molecules assuming an orientation parallel to GO layers (Figure 3). The schematic structural model composed using general geometric and chemical consideration provides an ≈10.5 Å interlayer distance for GO intercalated by one MB layer (Figure 4a). The structure of the close-packed layer similar to the one in solid MB was considered as the most likely type of intercalation (Figure 4b). MB is a flat molecule based on three carbon hexagons. The experimental change of the interlayer distance by 2.9 Å is only slightly smaller than the interlayer distance in graphite (≈3.3 Å) and allows one to suggest an almost homogeneous distribution of MB molecules in the GO structure. In the case of idealized close-packed MB layer intercalation, the formation of the GO/MB structure corresponds to the sorption of ≈320 mg of MB per gram of GO. The structure shown in Figure 4 is in reasonable agreement with experimental data considering a similar interlayer distance. A smaller experimental value for the sorption of MB (279 mg/g) indicates a somewhat diluted structure of the intercalated layer, i.e., the distance between neighboring molecules is somewhat larger than in the close-packed layer. It should be noted that both XRD and NR methods provide information about averaged values of the interlayer distance, while the analysis of the GO structure in the intercalated state is typically complicated by interstratification. Therefore, a small increase in the interlayer distance of GO films due to sorption of Rose bengal and crystal violet is likely explained by inhomogeneous intercalation. When a fraction of GO layers is intercalated with these dye molecules and the layers are randomly interstratified, the increased d-value reflects not the true change of the interlayer distance but only the proportion between the numbers of intercalated and nonintercalated layers.

It is interesting to compare the sorption of MB by thin films and by bulk graphite oxide. Therefore, sorption experiments were performed using powder graphite oxide exposed to MB solution using the same procedure as GO films. Intercalation of MB into graphite oxide resulted in an increase of *d*(001) from 7.9 Å for the pristine material up to 11.2 Å (Figure S6 in the SI). This value is in good agreement with the interlayer distance evaluated for MB-intercalated GO films using the NR method (10.8 Å). These values were recorded using air-dried materials, but the sorption actually occurs in ethanol solution. Therefore, we performed an XRD test in situ for the sample of graphite oxide immersed in an excess of MB solution. In this case, the *d*(001)=13.9 Å was measured. Therefore, the difference between *d*(001) in ethanol solution of MB and after air-drying was 2.8 Å. That is smaller than 4.9 Å increase of *d*(001) due to graphite oxide swelling in pure ethanol (7.8 and 12.7 Å in liquid ethanol and after drying, respectively). It can be concluded that intercalation of MB into the graphite oxide structure results in significant modification of the material swelling properties. In fact, graphite oxide and MB-intercalated graphite oxide need to be considered as two different materials. Our experiments also reveal that the sorption of MB is not completely reversible. The MB sorbed by graphite oxide could not be removed by washing in pure ethanol even after multiple cycles (Figure S8 in the SI).

Quantitative evaluation of MB sorption by bulk graphite oxide powder was performed using the standard UV absorption method. Graphite oxide powder was found to adsorb 214 mg/g of MB, which is somewhat smaller than the sorption found for the GO thin film (279 mg/g) using the NR method.

Note that sorption of MB by the thin film was measured after 1.5 h of immersion, while for the GO powder, 1 week of stirring in an excess amount of MB solution was used. Therefore, the difference cannot be related to the slower kinetic of sorption in thin films. Similar sorption of MB had been reported for dispersed GO in several earlier studies, typically performed in water solutions (220–240 mg/g).^40,44,47,51−53^ Some theoretical estimations provide maximal possible sorption of MB by the ideal GO structure on the level of 350 mg/g.^40^ A much higher sorption of MB was found for strongly defected GO with a small flake size and abundant holes.^45^

As discussed in our previous studies, the main difference between the structure of powder graphite oxide and GO laminates deposited from dispersions is in flake overlap. The GO flakes typically inherit the shape and layer packing from the parent graphite, which makes the surface of GO sheets in powder materials easily accessible for solvent and ions. The GO films and membranes are prepared by precipitation of single-layered flakes from water dispersions. The irregularly shaped GO flakes are randomly packed and overlapped in random orientations to form a strongly disordered layered structure. Therefore, solvent molecules and ions must diffuse along a zigzag pathway around the GO flakes to penetrate into the subsurface layers. The flake edges provide hindrances for the diffusion, and the swelling of GO laminates is often smaller compared to that of powders.^3,4,49^ It is expected that thicker multilayers will provide more hindrances for diffusion and a smaller overall sorption of, e.g., MB. Therefore, it was expected that sorption of MB by GO films and membranes will be slower compared to powders. It is somewhat surprising that the sorption of thin films is on the same level and even slightly higher compared to that of powders. However, the thin films studied in our experiments were composed of only 60–70 layers of GO, thus providing not that significant an increase in the diffusion pathway for penetration of solvent and solute molecules. In our experience, spin-coating becomes increasingly more difficult for preparation of thicker films due to the required multiple dropping of the dispersion on the substrate. However, it is very common to prepare freestanding GO membranes with few micrometer thickness using the vacuum filtration method.

### 3.2 Methylene Blue Sorption by GO Membranes

To verify the possible influence of multilayered GO thickness on the sorption of MB, we performed additional experiments with micrometer-thick freestanding membranes prepared by vacuum filtration. Vacuum filtration by definition suggests deposition over essentially not a flat surface of porous alumina filter, thus providing a material with a stronger disorder. Since the membranes are not suitable for NR experiments, the structural testing of MB blue sorption by GO membranes was limited to the XRD method and the bulk test of MB sorption.

Freestanding GO membranes with 6 μm thickness were immersed in the MB solution with the same concentration as graphite oxide powder and GO thin films prepared by spin-coating. The amount of sorbed MB was found using the UV absorption method. The membrane showed an MB sorption value of 128 mg/g, which is about half the value for thin films and powder graphite oxide. Note that 1 week of immersion time was used for membranes, similar to tests performed on powders.

**Figure 5:**
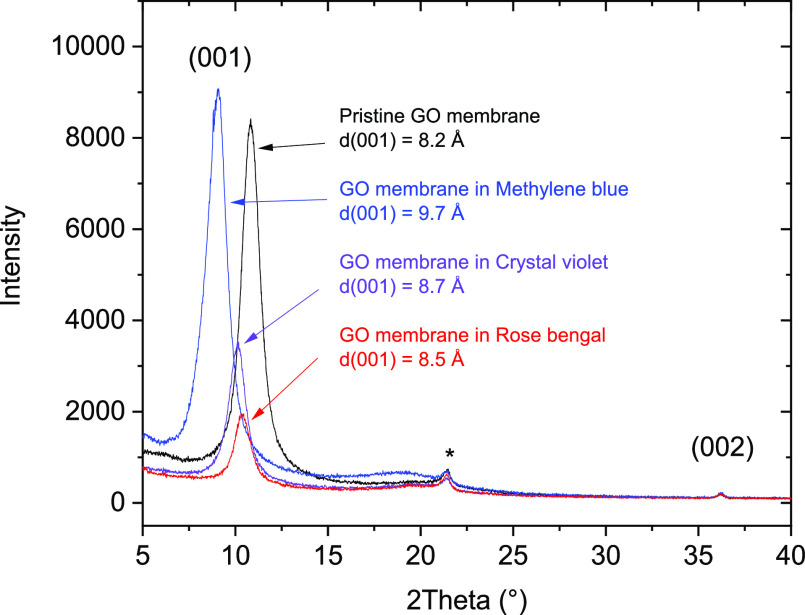
X-ray diffraction patterns recorded at ambient humidity from GO membranes after immersion in MB, CV, and RB ethanol (1 mg/mL) solutions for 2 weeks.

XRD patterns were recorded from air-dried GO membranes at ambient conditions and after immersion in MB, CV, and RB solutions followed by drying (Figure 5). The shape of *d*(001) reflections (Figure 5) is slightly asymmetric with the tail on the higher angle side, which is typical for GO materials. Asymmetric shape of XRD spots and diffuse scattering were observed in our previous XRD studies of GO membrane swelling.^3,4^ The numbers for *d*(001) represent the main component of this reflection.

In agreement with the smaller sorption of MB found by bulk sorption tests, the increase in the *d*(001) value due to MB sorption appeared to be significantly smaller compared to thin films, i.e., even after a much more prolonged exposure to the solution. An interlayer distance increase from 8.2 to 9.7 Å was observed for the GO film even after immersion in MB solution for two weeks. Note that the membranes were well-equilibrated with air conditions and the increase in the *d*(001) value is not due to the residual solvent. An even smaller increase in *d*(001) was found for GO membranes after exposure to RB and CV in the ethanol solution (0.3 and 0.5 Å, respectively). The increase in *d*(001) observed in GO membranes after exposure to the solution of dyes is smaller than the size of molecules, which is explained by the effects of random interstratification. Specific effects of interstratification in interpretation of XRD of GO materials are often misunderstood and need to be explained in more details

Interstratification is related to structures composed of mixed layers with different spacings, most often due to intercalation of some molecules. Random interstratification is common in layered hydrophilic materials^54−56^ and considered as a reason for gradual shifts in the *d*(001) position observed in GO upon intercalation by various molecules.^19^ The most common example of the effect explained by interstratification is the gradual change of *d*(001) of GO materials as a function of humidity and in temperature-dependent change of the swelling state in several liquid solvents.^3,26,57^ In this case, it is considered that GO interlayers hydrated (or solvated) with one or two layers of water (solvent) are randomly interstratified with nonhydrated layers. In our experiments, the random interstratification is related to layers of pristine (not intercalated) GO and GO layers intercalated with molecules of dyes. A single graphene oxide interlayer can also be intercalated inhomogeneously on the nanometer scale providing different distances between graphene oxide layers in intercalated and nonintercalated areas.^58^ The *d*(001) value provided by XRD is averaged over thousands of layers and averaged over variations in intercalation along each layer. Only one diffraction peak is observed for *d*(001) of GO due to the completely random stacking of layers, while the position of this reflection shifts depending on the proportion between the numbers of differently intercalated (or hydrated/solvated) layers.^59^ Therefore, small shifts of the *d*(001) value recorded for GO after immersion in solutions of CV and RB can be explained by the rather small proportion of intercalated layers in mostly nonintercalated mixtures of randomly interstratified structures.

The increase in *d*(001) due to the sorption of MB is smaller in GO membranes as compared to thin films and graphite powders, in agreement with the smaller MB sorption found by the bulk sorption test. Nevertheless, the chemical modification of the membrane by intercalation of MB is obvious and the value of sorption is significant and sufficient to affect membrane permeation tests.

The weight increase due to MB sorption in the GO membrane of about 13% and the increase in the interlayer distance of about 18% found in our experiments are significant. The change in the GO structure and removal of MB from solutions are likely to affect membrane permeation properties in several experimental setups. For example, experiments with diffusion of dyes between two compartments separated by relatively thick GO membranes were earlier performed using a rather low concentration of MB. The absence of MB diffusion from the compartment filled with the solution into the compartment with the pure solvent was assigned in these experiments to the size exclusion effect. That is, the MB “hydration radius” was considered too large relative to the size of permeation channels provided by the GO interlayer space.^37^ However, the high sorption capacity of the GO membrane to methylene blue was not taken into account in these studies.

**Figure 6:**
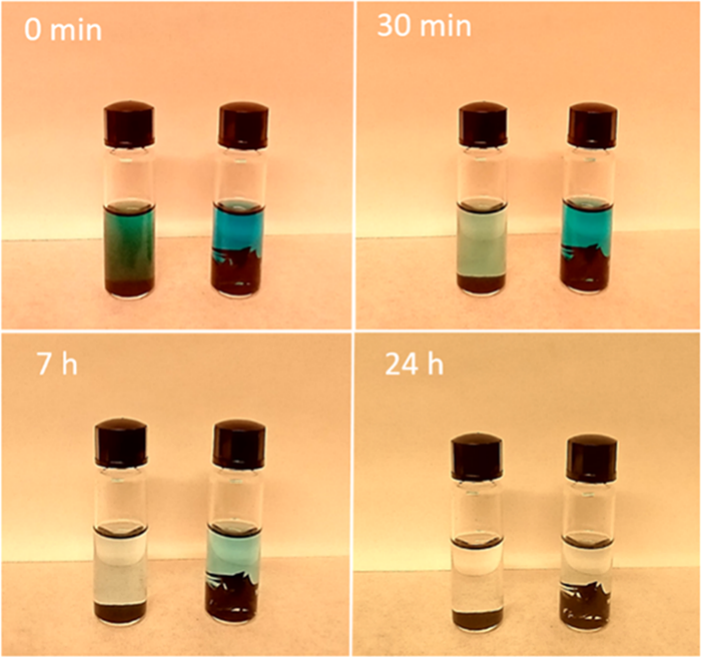
Sorption of methylene blue by Hummers graphite oxide powder (left vial) and by pieces of GO membrane (right vial), both samples with equal weight (3 mg) and 2 mL solution with concentration 20 mg/L. The original MB solution has a bright-blue color. The color disappears due to sorption by GO powders within tens of minutes, and for the GO membrane it occurs over a period of many hours.

To demonstrate this effect explicitly, several milligrams of GO powder and GO membranes were immersed into the ethanol solution of MB with concentration 20 mg/L, the same concentration as in permeation tests performed in ref (60). The sorption of MB can be followed by the change of solution color (Figure 6). Rapid sorption of MB by graphite oxide resulted in a change of solution color already after a few minutes. A completely colorless solution was observed after several hours of immersion. The piece of membrane immersed in the MB solution provides sufficient sorption capacity to cause complete disappearance of the typical blue color of the diluted MB solution but with slower kinetics. Almost complete discoloration of the solution was observed after 24 h. These results indicate that the properties of the similar GO membrane (both interlayer distance and composition) in permeation tests would be continuously changing over a period of hours and even days depending on the ratio between the weight of the membrane and the volume and concentration of the solution.

The permeation tests performed with GO membranes in refs^37, 60^ were based on measurements of dye concentrations on the feed and permeate sides of the membrane. The absence of detectable MB concentration on the permeate side was described as evidence of the size exclusion effect considering that the “solvation diameter” of ions (≈10 Å) is larger compared to the GO interlayer distance.

Our experiments demonstrate that MB intercalates into the GO structure with the molecule orientation parallel to GO layers. MB also partly fills the space between GO layers, thus changing the size of permeation channels. Moreover, sorption of MB is sufficiently strong to affect the concentration of solutions in the permeation experiments. The results of our sorption experiments (see also Figure 6) show that the concentration of MB in membrane filtration experiments had to decrease on the feed side, while MB penetrates into the interlayer space and intercalates the GO structure.

Notably, quite low concentrations of 20 mg/L were used earlier in permeation experiments with GO membranes.^36^ Considering the approximate weight of the GO membrane to be used in permeation experiments as 20 mg and concentration 20 mg/L, the sorption capacity of the membrane 15% by weight (≈3 mg) would be sufficient for complete removal of MB from a 150 mL feed volume. Significant decrease in concentration could be observed even for somewhat higher feed volumes. Therefore, no MB would be showing on the permeate side of membranes solely due to sorption, i.e., even if the membrane had holes. The MB would be removed from the solution even from the permeate side. The sorption effect is likely to be even stronger for thin GO films, as demonstrated by the twice higher sorption of MB by ≈500 Å films as compared to the 6 μm thick membrane. For very thin GO membranes,^37^ the effect of MB sorption will affect permeation properties mostly by the change in the effective size of permeation channels filled by intercalated molecules.

It can be concluded that methylene blue is not suitable for permeation tests aimed at establishing size exclusion effects related to the GO interlayer distance. The sorption of MB by the GO membrane significantly modifies both chemical composition and interlayer distance of GO. Intercalation also modifies the swelling of GO and changes the size of permeation channels. Experiments demonstrate that relatively large molecules easily penetrate into the GO interlayer space independent of their hydration radius if their shape is one- or two-dimensional. For example, GO membranes can be easily intercalated by long-chain alcohols inserted into the interlayer space parallel to oxidized graphene planes.^24,49^ It can be expected that many 2D molecules of larger size can also be intercalated into the GO structure in orientation parallel to planes.

## 4 Conclusions

In conclusion, sorption of methylene blue (MB), crystal violet (CV), and Rose bengal (RB) by thin GO films on Si support was quantitatively evaluated using the NR method. This method allows simultaneous estimation of the increase in film thickness and change in chemical composition after exposure of the GO film to ethanol solutions of dyes. Additional MB sorption tests were performed for bulk graphite oxide and freestanding micrometer-thick GO membranes. It is demonstrated that MB is not suitable for testing molecule size effects in permeation of GO membranes. Intercalation of MB into the GO structure occurs with significant expansion of the interlayer distance and significant sorption capacity (≈28% weight increase for thin films and ≈13% for the membrane). The structure intercalated with the layer of MB needs to be considered as the chemically modified form of GO with different properties. In contrast to the sorption of MB, GO is not easily intercalated by CV and RB dyes in ethanol solution. This effect is assigned to significant differences in the geometry of the dye molecules. The flat-shaped MB molecule can be easily inserted between GO layers. The twisted and essentially not flat shape of CV and RB prevents their intercalation. It can be concluded that the hydration radius of molecules is not the key parameter for penetration of molecules into the GO interlayer space. Relatively large molecules (e.g., MB) can be intercalated into the GO structure if their fat shape orientation parallel to planes and thickness of the layer allows penetration into GO interlayers swollen in the polar solvent (such as ethanol).

## Supporting Information Available

Additional NR data and calculations as well as XRD measurements for the reversibility test and swelling properties of GO intercalated with MB (PDF)
