# Supplementary material for: Intercalation of Dyes in Graphene Oxide Thin Films and Membranes
Source: J Phys Chem C Nanomater Interfaces. 2021 Mar 23;125(12):6877–85. doi: 10.1021/acs.jpcc.1c00327 (PMC8043058; doi:10.1021/acs.jpcc.1c00327)
Supplement: Supplementary file 1 — jp1c00327_si_001.pdf [file jp1c00327_si_001.pdf]

Supporting information

Intercalation of Dyes in Graphene Oxide Thin Films and Membranes

Andreas Nordenström,[a] Nicolas Boulanger,[a] Artem Iakunkov,[a] Igor Baburin,[b] Alexey Klechikov,[c] Alexei Vorobiev,[c] Alexandr Talyzin[a]*

[a.] Department of Physics, Umeå University, S-90187, Umeå, Sweden.

[b.] Theoretische Chemie, Technische Universitat Dresden, Bergstraße 66b, 01062 Dresden, Germany

[c.] Department of Physics and Astronomy, Uppsala University, Uppsala 751 20, Sweden

*Corresponding author. . Tel: 090-786 63 20. Email: alexandr.talyzin@umu.se

1. Introduction of parameters, constant and simple relations used for modelling of neutron reflectivity data

1.1 Nomenclature

ρ – scattering length density (SLD) obtained directly from the fit of the neutron reflectivity curve;

L – thickness of the GO film obtained directly from the fit of the neutron reflectivity curve;

d = 7.9 Å – spacing between GO monolayers in the direction perpendicular to the film surface as confirmed by XRD measurements;

N – number of GO monolayers in the sample film;

S_H = 5.246 Å^2 – area of the carbon hexagon, which is assumed to be constant.

b – neutron scattering length (NSL) specific for every isotope (see Table S1);

B = $\sum b_i$ – total NSL of molecules or crystal unit cells consisting of several atoms with corresponding individual b_i (see Table S1);

V – volume of the GO unit cell.

Table S1. NSL (B) values for relevant chemical elements and molecules (Source: https://www.ncnr.nist.gov/resources/n-lengths/).

**Compound**	**Formula**	**B(10^{-4}Å)**
Carbon	C	0,6646
Oxygen	O	0,5803
Hydrogen	H	-0,3739
Chlorine	Cl	0,9577
Nitrogen	N	0,936
Sulphur	S	0,2847
Iodine	I	0,528
Sodium	Na	0,363
Water	H_2O	-0,1675
Methylene Blue	$C_{16}H_{18}ClN_3S$	7,9538
Crystal Violet	$C_{25}H_{30}ClN_3$	9,1637
Rose Bengal	$C_{20}H_2Cl_4I_4Na_2O_5$	22,1145

1.2 Equations

$V = d \cdot S_H = L \cdot S_H / N$ (1)

$N = L/d$ (2)

$B = \rho \cdot V$ (3)

1.3 Calculation of ambient state composition of GO film

Experimental values found for ambient state of GO films are presented in Table S2. V, N and B was calculated using Equation 1,2 and 3 respectively. Note that the unit cell volume will be the same for all films since the interlayer spacing is the same.

Table S2. Experimentally obtained values for the GO films in ambient state.

ID	L_0 (Å)	ρ_0 (10^{-6} Å^{-2})	d (Å)	V (Å^3)	N	B_0 (10^{-4}Å)
Film A	501	3.84	7.9	41.4	63,4	1.59
Film B	530	3.90	7.9	41.4	67,1	1.62
Film C	380	3.88	7.9	41.4	48,1	1.61

Assuming that the GO unit cell has composition $C_2O_{0.8}H_{0.24}$ it can be found that the corresponding NSL is B = $1.704 \cdot 10^{-4}$ Å. Notably this value is higher than the experimental values presented in Table S2. To eliminate this contradiction we must consider water molecules as part of the GO unit cell. The number of water molecules per formula unit of GO will be $N_{Water} = (B - B_0)/B_{Water}$. Thus the ground state of the films will be as shown in Table S3. The ground state as shown in Table S3 is used to determine the sorption of various dyes.

Table S3. Ground state of GO films.

ID	B-B_0 (10^{-5}Å)	N_{Water}	Ground state
Film A	1.13	0.67	$C_2O_{0.8}H_{0.24} + (H_2O)_{0.67}$
Film B	0.88	0.52	$C_2O_{0.8}H_{0.24} + (H_2O)_{0.52}$
Film C	0.96	0.57	$C_2O_{0.8}H_{0.24} + (H_2O)_{0.57}$

1.4 Calculating the number of adsorbed molecules by studying the change in L and ρ

When GO film is intercalated with some molecule x the chemical composition of the film will change. If the NSL (B) of the molecule is sufficiently different from the GO film there will be an observable difference in SLD (ρ). In addition, the film thickness (L) will increase because of the intercalated molecules. By studying how ρ and L change, it is possible to determine the number of adsorbed molecules. Let L_0 and ρ_0 to be the experimentally obtained values for the GO film in ground state and let L_x and ρ_x be the values after sorption of some molecule x. It is reasonable to assume that the number of monolayers in the film remains constant after sorption and hence the new interlayer distance will be $d_x = L_x/N$. We can also calculate the unit cell volume (V_x) by inserting d_x into Eq. 1. The new NSL (B_x) can be calculated using ρ_x and V_x in Eq. 3. The number of adsorbed molecules can then be determined by studying the change in B from the ground state of the GO film as $N_{Molecule} = (B_x - B_{Ground\ state})/B_{Molecule}$, where $B_{Molecule}$ is the NSL of the intercalated molecule. This answer is trivial and absolutely decisive when only one type of molecule intercalates. Note that it is also possible to recalculate $N_{Molecule}$ to grams of adsorbed molecule per gram of GO.

2. Neutron reflectivity data

2.1 GO film before and after MB sorption from ethanol solution.

Table S4. Neutron reflectivity data for Film A in ground state and after exposure to Methylene Blue.

**Film A ground state**		
SLD	3,84E-06	Å-2
Thickness	501	Å
b_0	1,59E-04	Å
N	63,4	st
$b_{0,ideal}$ - b_0	1,13E-05	Å
N water per f.u.	0,672	st
Corrected GO formula	$C_2O_{0.8}H_{0.24}$ + $(H_2O)_{0.672}$	
Corrected GO unit cell weight	49,1704759	g/mol
**Film A after Methylene Blue exposure**		
SLD	3,42E-06	$Å^{-2}$
Thickness	683	Å
d	10,77	Å
V	56,50	$Å^2$
b	1,93E-04	Å
Change in b from ground state	3,41E-05	Å
Number of molecules per f.u.	0,0429	st
Corresponding weight	278,79	mg / g of GO

The data presented above were obtained using fitting NR curves by two layers. Additional fitting was performed also using three layers adding interface between Si and GO films and fixing roughness of Si substrate. Figure S1 shows fitting curves and SLD profiles of GO films before and after MB sorption. The SLD and thickness determined using 3 layer fitting model are very similar to the two layer model.

The thickness change due to MB sorption is 178Å in three layer model compared to 182Å in two layer model, SLD change in 3 layer model from 3.86 to 3.42 whereas in 2 layer model the change is from 4.84 to 3.41. It can be concluded that the choice of model is not affecting (within errors) final results in terms of film thickness change and amount of sorbed MB. The details of SLD profile shape are not analyzed in this study.

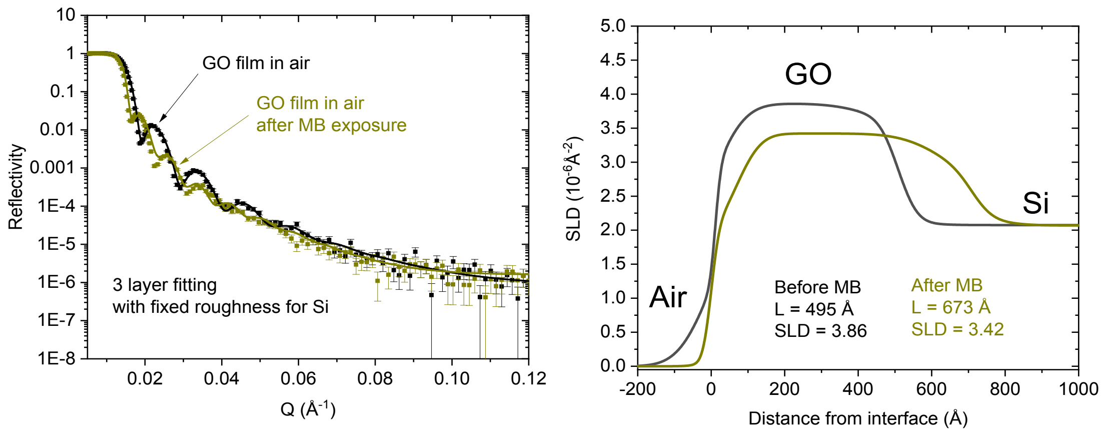

Figure S1. Fitting of NR data using 3 –layer model a) Neutron reflectivity data (including error bars) obtained from GO film in ambient conditions before and after Methylene Blue sorption b) Scattering length density profile of the GO film before and after RB sorption obtained as result of the modelling (fitting) of experimental data.

2.2 GO film before and after RB sorption from ethanol solution.

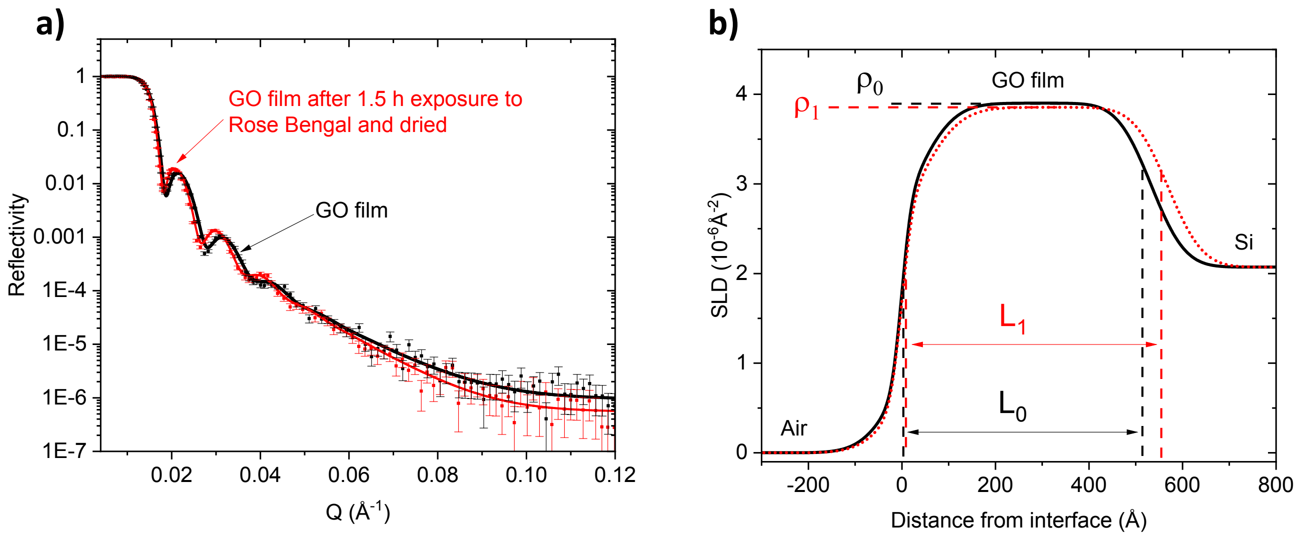

Figure S2. a) Neutron reflectivity data (including error bars) obtained from GO film in ambient conditions before and after Rose Bengal sorption b) Scattering length density profile of the GO film before and after RB sorption obtained as result of the modelling (fitting) of experimental data.

Table S5. Neutron reflectivity data for Film B in ground state and after exposure to Rose Bengal.

**Film B ground state**		
SLD	3,90E-06	Å^{-2}
Thickness	530	Å
b_0	1,62E-04	Å
N	67,1	st
$b_{0,ideal}$ - b_0	1,13E-05	Å
N water per f.u.	0,524	st
Corrected GO formula	$C_2O_{0.8}H_{0.24} + (H_2O)_{0.524}$	
Corrected GO unit cell weight	46,496048	g/mol
**Film B after Rose Bengal exposure**		
SLD	3,85E-06	Å^{-2}
Thickness	564	Å
d	8,41	Å
V	44,10	Å^{2}
b	1,70E-04	Å
Change in b from ground state	8,16E-06	Å
Number of molecules per f.u.	0,0037	st
Corresponding weight	80,794	mg / g of GO

2.3 GO film before and after CV sorption from ethanol solution.

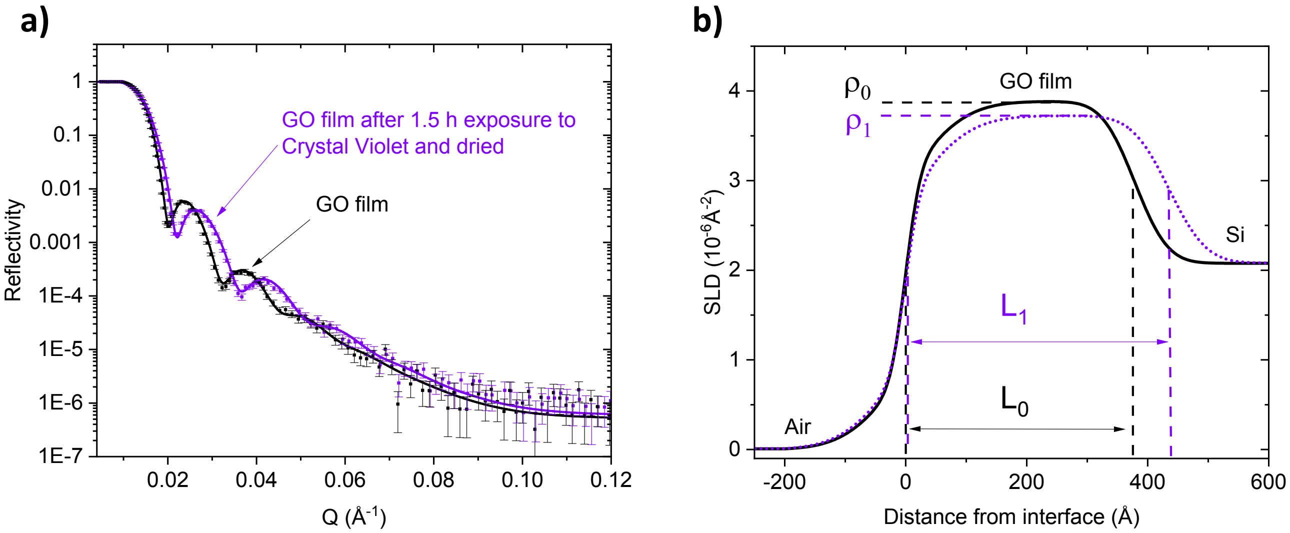

Figure S3. a) Neutron reflectivity data (including error bars) obtained from GO film in ambient conditions before and after CV sorption b) Scattering length density profile of the GO film before and after CV sorption obtained as result of the modelling (fitting) of experimental data

Table S6. Neutron reflectivity data for Film B in ground state and after exposure to Crystal Violet.

**Film C ground state**		
SLD	3,88E-06	$Å^{-2}$
Thickness	380	Å
b_0	1,61E-04	Å
N	48,1012658	st
$b_{0,ideal}$ - b_0	1,1257E-05	Å
N water per f.u.	0,573	st
Corrected GO formula	$C_2O_{0.8}H_{0.24}$ + $(H_2O)_{0.573}$	
Corrected GO unit cell weight	47,3875239	g/mol
**Film C after Crystal Violet exposure**		
SLD	3,72E-06	$Å^{-2}$
Thickness	436	Å
d	9,06	Å
V	47,551	$Å^2$
b	1,77E-04	Å
Change in b from ground state	1,6089E-05	Å
Number of molecules per f.u.	0,0176	st
Corresponding weight	151,164	mg / g of GO

2.4 Reference experiment with GO film and pure ethanol.

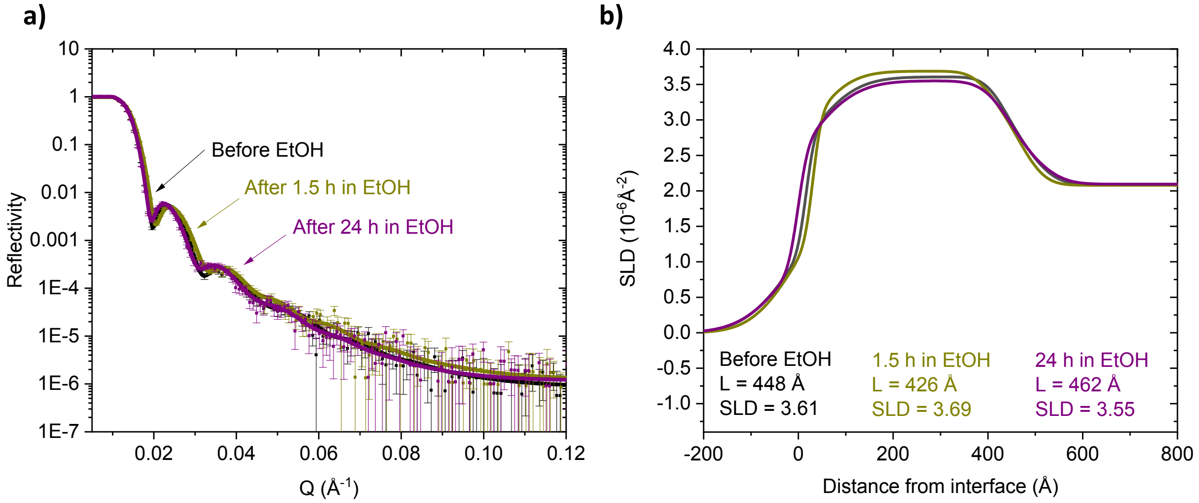

Figure S4. NR data for GO film before (black), after 1.5 h (green) and 24 h (purple) immersion in pure ethanol followed by air drying. a) Neutron reflectivity data b) Scattering length density profiles.

GO is known to swell reversibly in ethanol vapor and in liquid ethanol. The reference experiment with pure ethanol was performed in order to verify the stability of GO films subjected to certain turbulence when the substrate is immersed into ethanol and removed from ethanol. The fitting of NR curves shown in Figure S3 resulted in GO film thickness values 448 Å (before ethanol), 426 Å after 1.5 h immersion and 462 Å after 24 h immersion. The change of film thickness relative to reference pristine film corresponds approximate to removal or addition of two GO layers. Assuming that the same decrease of thickness occurs in Films 1-3 studied for sorption of dyes, the value of d-spacings need to be considered with error ± 0.3 Å.

The film thickness is affected by random variations of air humidity in the experimental hall which is estimated to result in ±0.3 Å error in d-spacing values. In principle, the film thickness can be slightly changing due to slight re-stacking of GO flakes in process of ethanol swelling and evaporation, e.g. due to flattening or appearance of some wrinkles. However, humidity effect is more likely reason for observed variations of film thickness since the same film showed first small decrease (after 1.5 h immersion) and after second immersion for 24 hours small increase of thickness) and in agreement with SLD value change.

3. XPS of precursor GO

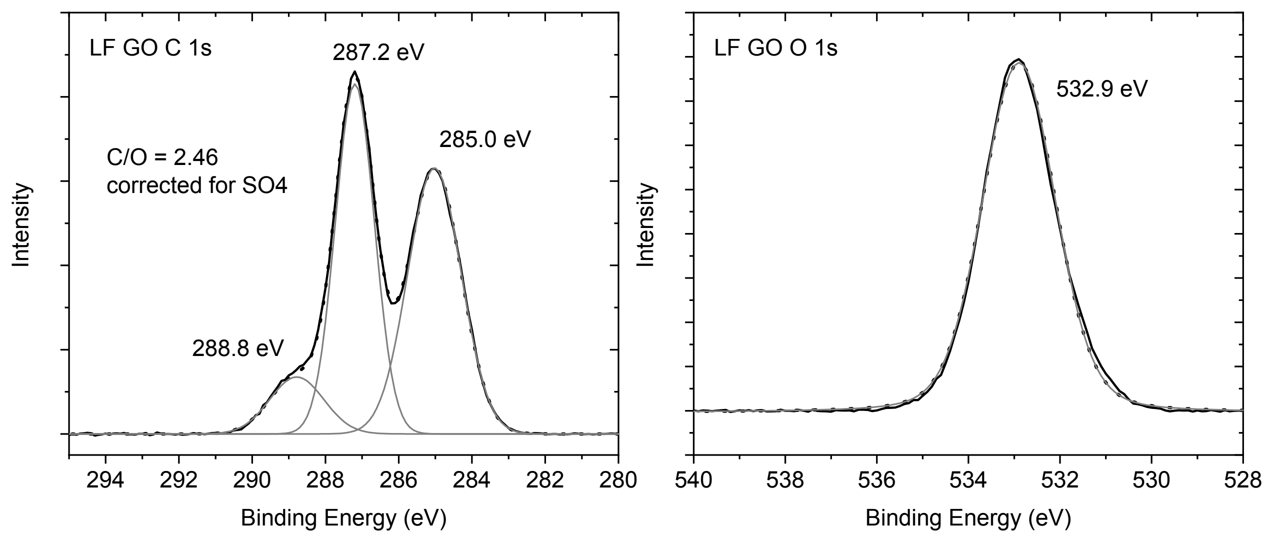

Figure S5. XPS spectra recorded from precursor graphite oxide, C1s and O1s parts.

4. Additional XRD data

4.1 XRD of GO powder exposed to ethanol solution of Methylene Blue

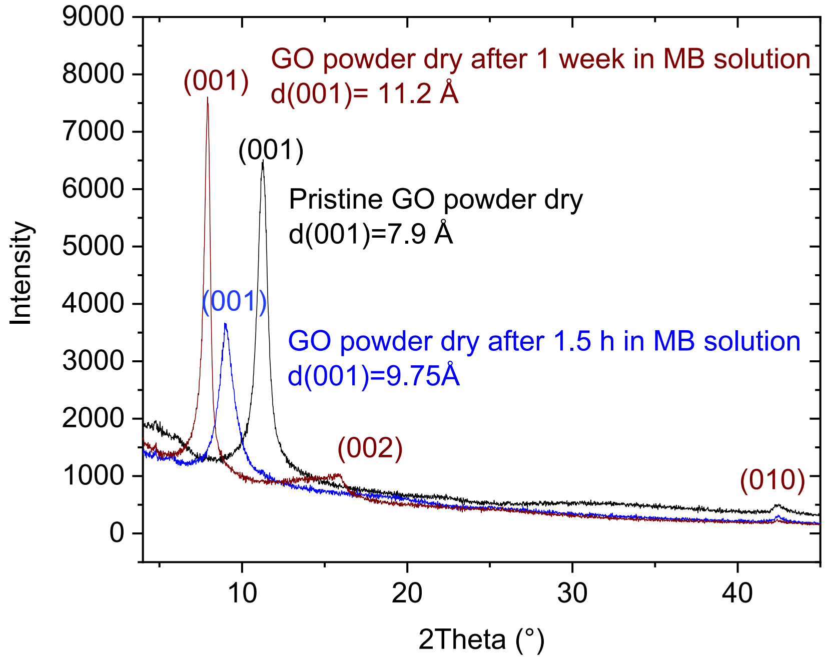

Figure S6. XRD patterns recorded from pristine GO powder, powder exposed to ethanol MB solution for 1.5 hours and powder GO exposed to ethanol MB solution for 1 week with stirring.

4.2. Swelling test of GO intercalated with Methylene Blue

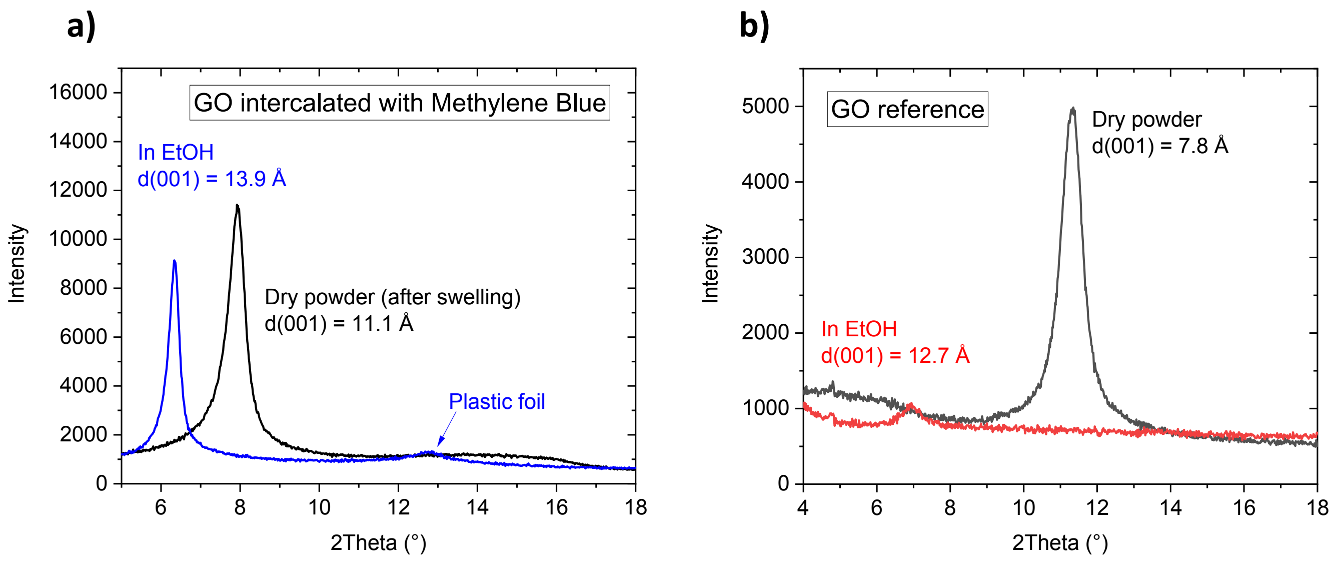

Figure S7. a) GO powder intercalated with Methylene Blue dry and in EtOH, b) Precursor GO powder dry and in EtOH. As can be seen the swelling is not quite as strong for GO intercalated with MB.

4.3. XRD of GO powder exposed to Methylene Blue and washed with ethanol to verify reversibility of sorption.

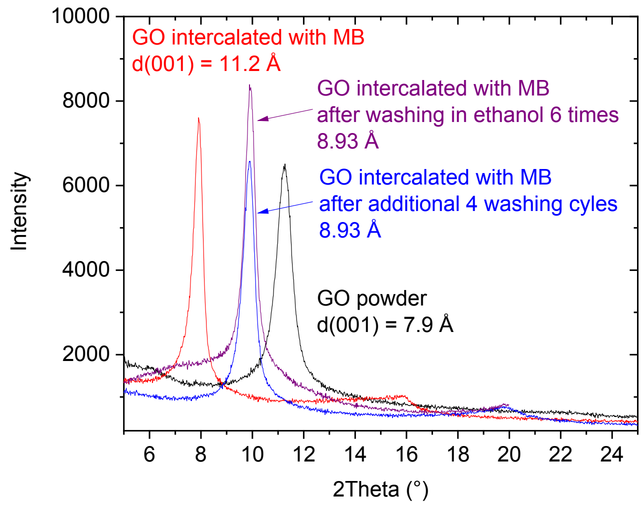

Figure S8. GO powder intercalated with Methylene Blue and washed with excess of pure ethanol in 6 cycles (blue) and after additional 4 washing cycles (purple). The data demonstrate that part of the MB is intercalated irreversibly and can not be removed even after prolonged washing. The washing was continued until no color was visible in added ethanol.
